# Exploring the photocatalytic and photodynamic effects of BODIPY-linked titanium dioxide nanoparticles

**DOI:** 10.55730/1300-0527.3623

**Published:** 2023-09-30

**Authors:** Seda DEMİREL TOPEL

**Affiliations:** Department of Electrical-Electronics Engineering, Faculty of Engineering and Natural Sciences, Antalya Bilim University, Antalya, Turkiye

**Keywords:** Titanium dioxide, photocatalyst, BODIPY, photosensitizer, photodynamic therapy

## Abstract

This study aimed to investigate the photocatalytic and photodynamic actions of a 2,6-diiodinated-BODIPY (2,6-diI_2_-Bod) photosensitizer (PS) covalently linked to TiO_2_ nanoparticles (NPs). The TiO_2_ NPs were synthesized utilizing the hydrothermal technique, yielding an average particle size of 4–5 nm as ascertained by transmission electron microscopy and 6.4 nm as determined by dynamic light scattering measurements. The integration of the nonsoluble Bod PS to TiO_2_ NPs provided increased dispersibility in physiological media, making it more applicable to photodynamic therapy applications. Bod-TiO_2_ NPs exhibited moderate efficiency of 56.9% in singlet oxygen generation. Additionally, the photocatalytic behavior of the TiO_2_ NPs was evaluated using methylene blue and 83.8% photodegradation efficiency was observed. The loading efficiency and loading content of the Bod PS were found to be 90.8% and 54.5%, respectively. These results suggest that Bod-TiO_2_ NPs possess higher dispersibility in aqueous solutions, moderate photocatalytic activity, and potential for photodynamic therapy applications.

## 1. Introduction

The intensive study of photodynamic therapy (PDT) in recent years has been devoted to exploring novel photosensitizers (PSs) and proper carriers for their delivery. In this approach, dyes and nanostructures have been integrated into a single system to enhance the selectivity and efficiency of the PS in therapy [1]. In this context, the nanostructures encompass a broad range of entities, including liposomes, lipid micelles, niosomes, dendrimers, polymeric NPs, quantum dots (QDs), fullerenes, cubosomes, and metallic nanoparticles (NPs). Particular attention should be given to the latter type, such as Fe_2_O_3_, AgO_2_, CuO, ZnO, and TiO_2_ NPs [2]. Notably, modifying drug molecules with these NPs may enhance their targeting in diseased tissues and improve their penetration abilities through cells [3]. In the present study, TiO_2_ was chosen as the metallic NP to be integrated with a synthesized BODIPY PS. The reasons for selecting TiO_2_ NPs include low toxicity, biocompatibility, and chemical stability [4,5]. Studies have also shown that these NPs exhibit rapid binding to cancer cells with acidic microenvironments [6]. Besides these advantageous properties, the primary reason for preferring TiO_2_ NPs in this study is their excellent photocatalytic behavior under ultraviolet (UV) excitation, which produces reactive oxygen species (ROS), facilitating the disruption of cancer cells [7]. The mechanism for the production of ROS relies on the generation of an electron pair (e^−^) and a positively charged hole (h^+^) after the UV irradiation of the TiO_2_ NPs. These photogenerated species can be trapped by the surface of the particle and further interact with electron acceptors (e.g., molecular oxygen) and donors (e.g., H_2_O molecules) to produce superoxide anions (O_2_^•−^), hydroxyl radicals (HO^•^), and, in some cases, singlet oxygen (^1^O_2_) [6,7]. In the presence of these radicals and active species, TiO_2_ plays an essential role in photocatalyst applications and paves the way for new applications of PDT [4].

PDT is an alternative cancer treatment technique that involves combining a light-sensitive molecule, known as the PS, with appropriate light at a specific wavelength suitable for activating the PS molecule [8]. When the light interacts with the PS, the PS molecule becomes excited to higher energy states and transfers its energy to a long-lived triplet excited state (T_1_) through intersystem crossing. The energy in the T_1_ state is then further transferred to molecular oxygen, forming ROS, which subsequently destroy the cancer cells [8]. In general, hematoporphyrin derivative (HpD), PHOTOFRIN, and PHOTOGEM are known as first-generation PSs; 5-aminolevulinic acid (ALA), chlorines such as meta-tetra(hydroxyphenyl)chlorin (m-THPC), pheophorbides, texaphyrins, and phthalocyanines are known as second-generation PSs; and some ruthenium-based PSs (e.g., Ru(II) tris-bipyridine) and VISUDYNE (PS-encapsulated liposomes) are known as third-generation PSs. All of these are considered effective PS agents for generating ^1^O_2_ [9]. Besides these types of PSs, the heavy atom-incorporated BODIPY (boron-dipyrromethene) molecule is also known for its excellent ^1^O_2_ generation under proper light excitation [10–13]. Additionally, BODIPY exhibits outstanding photophysical properties, including a high fluorescence quantum yield (*φ* > 70%), a high absorption coefficient (*ɛ* > 50,000), intense fluorescence emission in the range of 510–800 nm, and high photostability [14,15].

In recent years, BODIPY PSs have been integrated into various nanosystems, such as liposomes, metallic NPs, polymeric NPs, QDs, and dendrimers, which can be considered as third-generation PSs. This integration enhances their solubility and usability in aqueous solutions for PDT [16–18]. For instance, Chang et al. synthesized a PEGylated 2,6-diBr_2_-BODIPY PS and encapsulated it with doxorubicin, then investigated both the chemo- and photodynamic therapy effects [19]. PEG was used as an anionic polymeric carrier agent to increase the solubility of the PS. These authors observed a time-dependent decrease in the absorbance intensity of diphenylisobenzofuran (DPBF) when it was used as a ^1^O_2_ trap molecule in the presence of the PS under light excitation of 450 nm. On the other hand, doxorubicin exhibited a mildly pH-dependent release (pH 5.0 and 6.0), which indicated that the PS/PEG carrier system acted as both a chemo- and photodynamic therapy agent [19]. Mangalath et al. developed BODIPY-conjugated graphene oxide quantum dots, which resulted in high water solubility and an excellent ^1^O_2_ generation yield (90%) [20]. Prieto-Montero et al. prepared BODIPY PS-conjugated mesoporous SiO_2_ NPs and further functionalized them with PEG and folic acid (FA) to increase their solubility in water [21]. They revealed that the PS-SiO_2_/PEG/FA system demonstrated promising ^1^O_2_ efficiency on HeLa cells in PDT [21]. Apart from these nanocarriers, bare TiO_2_ NPs can act as both carrier agents and PSs due to their photocatalytic behavior [4]. There are reports available on TiO_2_ hybrid nanostructures, such as graphene oxide functionalized TiO_2_ NPs, TiO_2_-coated upconversion NPs [22], nitrogen-doped TiO_2_ NPs [23], PVP-PEG-assisted Fe_2_O_3_-TiO_2_ NPs [24], FA-functionalized TiO_2_ NPs [25], and phthalocyanine-conjugated TiO_2_ NPs, all tailored for PDT applications [26]. However, to the best of our knowledge, there have been no reports on the synthesis of 2,6-diI_2_-BODIPY PS conjugated to TiO_2_ NPs and the investigation of their PDT and photocatalytic effects.

In this study, it was hypothesized that integrating the 2,6-diI_2_-Bod PS molecule with TiO_2_ would enhance its ^1^O_2_ generation and dispersibility in water, making it suitable for use as a PDT agent. In addition to investigating their ^1^O_2_ generation and photocatalytic behaviors, the characterization of the TiO_2_ NPs and the photophysical properties of the Bod-TiO_2_ hybrid NPs were studied systematically.

## 2. Materials and methods

### 2.1. Materials

All reagents, including *p*-hydroxybenzaldehyde, benzo-18-crown-6, 6-bromohexanoic acid, 2,4-dimethylpyrrole, trifluoroacetic acid (TFA), *p*-chloranil, triethylamine (TEA), boron trifluoride diethyl etherate (BF_3_.OEt_2_), iodic acid (HIO_3_), potassium carbonate, sodium sulfate (Na_2_SO_4_), titanium(IV) isopropoxide (Ti(OPri)_4_), polyethylene imine (PEI; branched, molecular weight 25,000 g/mol), 4-dimethylaminopyridine (DMAP), and *N,N′*-dicyclohexylcarbodiimide (DCC), were obtained from Sigma-Aldrich (St. Louis, MO, USA) in a state of high purity, with a minimum purity level of 99.5%. Solvents of analytical grade were purchased from Sigma-Aldrich and utilized without undergoing any purification processes. Silica gel 60 F254 plates (Merck, Darmstadt, Germany) were utilized for monitoring the reaction process through thin-layer chromatography (TLC). To purify both the PS intermediate and the resulting products, flash silica gel column chromatography was conducted using silica gel 60 (Merck; particle size: 0.040–0.063 mm, 230–400 mesh ASTM).

^1^H NMR and ^13^C NMR spectra were acquired using a DPX-400 device (Bruker, Billerica, MA, USA) in CDCl_3_ solvent with TMS as the internal standard. Mass spectra were obtained using the 6224 TOF LC/MS and 6530 Accurate Mass Q-TOF LC/MS (Agilent Technologies, Santa Clara, CA, USA). Fourier transform infrared (FTIR) spectrometry was employed to confirm the functional groups present in both the TiO_2_ NPs and the PS molecule using the L160000R analyzer system (PerkinElmer, Waltham, MA, USA) over a wavelength range of 4000 to 400 cm^−1^. The size and morphology of the TiO_2_ NPs were determined using a transmission electron microscope (TEM; Zeiss, Oberkochen, Germany). The Zetasizer Nano ZS (Malvern PANalytical, Malvern, UK) was utilized to measure the size distribution of the TiO_2_ NPs in water through dynamic light scattering (DLS). For experiments involving singlet oxygen, DPBF purchased from Sigma-Aldrich was used as a trap molecule. A PerkinElmer Lambda 650 UV-Vis spectrophotometer was used to observe the singlet oxygen generation. The photophysical properties of the PS and hybrid materials were determined using a fluorescence spectrometer (Agilent Technologies, Cary Eclipse model) and UV-Vis spectrometer (Agilent Technologies, Cary 100 model).

### 2.2. Methods

**Figure 1:**
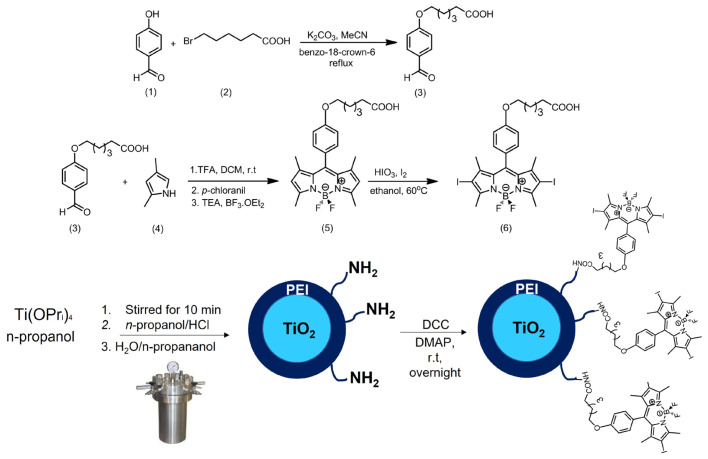
Synthesis scheme of the 2,6-diI_2_-BODIPY (**6**) and 2,6-diI_2_-BODIPY/TiO_2_ NPs.

The schematic depiction of the synthesis process of the BODIPY PS and its linkage to TiO_2_ is presented in Figure 1. To design a functional BODIPY molecule for PDT, two crucial substitutions on the BODIPY scaffold are essential. First, the formation of a carboxylic acid functional group within the BODIPY molecule is required, providing a binding site suitable for amine-decorated TiO_2_ NPs, while the second modification involves introducing iodine groups at the 2,6-positions of the BODIPY molecule. This modification enhances the production of singlet oxygen by facilitating intersystem crossing.

#### 2.2.1. Synthesis of 6-(4-formylphenoxy)hexanoic acid (3)

The product was obtained according to our previously described method [27]. *p-*Hydroxybenzaldehyde (8.18 mmol) was dissolved in dry acetonitrile (20 mL) in a 250-mL round-bottom flask. To the first solution, K_2_CO_3_ (49.1 mmol) and benzo-18-crown-6 (0.08 mmol) were added. To this mixture, 6-bromohexanoic acid (12.3 mmol) dissolved in dry acetonitrile (15 mL) was added. The reaction mixture was refluxed for 24 h. The product formed as a white precipitate in the reaction mixture. The crude product was separated by filtering the reaction mixture and was washed twice with cold dichloromethane (DCM). It was then dissolved in distilled H_2_O (15 mL). The solution was neutralized using 4 M HCl (37%). White precipitates were formed in the solution. The solid was filtered off and dried in a vacuum oven at 40 °C. The white product (**3**) was obtained in a 58% yield. ^1^H NMR (400 MHz, CDCl_3_) δ ppm: 9.49 (s, 1H), 7.51 (d, 2H, *J =* 8.4 Hz), 6.70 (d, 2H, *J =* 8.4 Hz), 3.75 (t, 2H, *J =* 6.3 Hz), 2.01 (t, 2H, *J =* 7.2 Hz), 1.56–1.49 (m, 2H), 1.41–1.34 (m, 2H), 1.25–1.19 (m, 2H) (Supporting Information, Figure S1); ^13^C NMR (100 MHz, CDCl_3_) δ ppm: 195.1 (C=O), 176.5 (COOH), 166.0 (C_ipso_), 136.0 (CH), 130.0 (C_ipso_), 118.7 (CH), 72.0 (CH_2_), 37.8 (CH_2_), 32.5 (CH_2_), 29.4 (CH_2_), 28.4 (CH_2_) (Supporting Information, Figure S2).

#### 2.2.2. Synthesis of 1,3,5,7-tetramethyl-8-[4-(5-carboxypentyloxy)phenyl]-4,4-difluoro-4-bora-3a,4a-diaza-s-indacene (5)

DCM (200 mL) was placed in a round-bottom flask with capacity of 250 mL and degassed under nitrogen atmosphere for 15 min. Compound **3** (2.96 mmol) was dissolved in the degassed DCM. To the first solution, 2.4-dimethylpyrrole (5.92 mmol) was added, followed by 3 drops of TFA, and the mixture was stirred overnight at room temperature. Afterward, *p*-chloranil (3.26 mmol) was added and stirred for 5 h. TEA (5 mL) and BF_3_.OEt_2_ (5 mL) were then added and stirred for an additional 30 min. The reaction mixture was subjected to three extractions with salt-saturated water (100 mL), followed by drying of the organic phase over Na_2_SO_4_. Subsequently, the solvent was evaporated under reduced pressure. The crude product was purified using a CHCl_3_:MeOH (3%) eluent system through flash column chromatography. The orange product was obtained in a 27% yield. ^1^H NMR (400 MHz, CDCl_3_) δ ppm: 7.16 (d, 2H, *J* = 8.6 Hz, Ar-CH) and 7.00 (d, 2H, *J* = 8.6 Hz, Ar-CH), 5.99 (s, 2H, CH), 4.03 (t, 2H, *J* = 6.4 Hz, CH_2_), 2.56 (s, 6H, CH_3_), 2.44 (t, 2H, *J* = 7.4 Hz, CH_2_), 1.89–1.84 (m, 2H, CH_2_), 1.78–1.73 (m, 2H, CH_2_), 1.63–1.57 (m, 2H, CH_2_), 1.45 (s, 6H, CH_3_) (Supporting Information, Figure S3); ^13^C NMR (100 MHz, CDCl_3_) δ ppm: 179.0 (COOH), 159.6 (C_ipso_), 155.2 (C_ipso_), 143.2 (C_ipso_), 141.9 (C_ipso_), 131.9 (C_ipso_), 129.2 (CH), 121.1 (CH), 115.1 (CH), 67.7 (CH_2_), 33.9 (CH_3_), 28.9 (CH_2_), 25.6 (CH_2_), 24.5 (CH_2_), 14.6 (CH_3_), 1.42 (CH_2_) (Supporting Information, Figure S4).

#### 2.2.3. Synthesis of 2,6-diI_2_-BODIPY (6)

A solution of compound **5** (1.10 mmol) in ethanol (200 mL) was heated to 60 °C after adding I_2_ (2.31 mmol). Following this, HIO_3_ (2.75 mmol) was added to the mixture and it was further heated at 60 °C for 1.5 h. The reaction progress was monitored using TLC. After the completion of the reaction, the ethanol was evaporated under reduced pressure. The resulting product was dissolved in DCM and then subjected to three extractions with sodium thiosulfate-saturated water (100 mL). The organic phase was dried using Na_2_SO_4_ and the solvent was evaporated under reduced pressure. Finally, the crude product was purified using a CHCl_3_:MeOH (3%) eluent system through flash column chromatography. The pink-colored product was obtained in 42% yield. ^1^H NMR (400 MHz, CDCl_3_) δ ppm: 7.03 (d, 2H, *J* = 8.4 Hz, Ar-CH), 6.93 (d, 2H, *J* = 8.4 Hz, Ar-CH), 3.94 (t, 2H, *J* = 6.4 Hz, CH_2_), 2.54 (s, 6H, CH_3_), 2.29 (t, 2H, *J* = 7.4 Hz, CH_2_), 1.81–1.72 (m, 2H, CH_2_), 1.69–1.61 (m, 2H, CH_2_) and 1.52–1.50 (m, 2H, CH_2_), 1.35 (s, 6H, CH_3_) (Supporting Information, Figure S5); ^13^C NMR (100 MHz, CDCl_3_) δ ppm: 160.0 (C_ipso_), 156.5 (C_ipso_), 145.4 (C_ipso_), 141.7 (C_ipso_), 131.7 (C_ipso_), 128.9 (C_ipso_), 126.5 (C_ipso_), 129.0 (CH), 115.4 (CH), 67.9 (CH_2_), 33.8 (CH_3_), 28.9 (CH_2_), 25.6 (CH_2_), 25.0 (CH_2_), 17.2 (CH_3_), 16.0 (CH_2_) (Supporting Information, Figure S6). ESI MS (m/z): (C_25_H_27_BF_2_I_2_N_2_O_3_) [M-H]^−^ calculated as 705.0111, found as 705.0172 (Supporting Information, Figure S7)

### 2.3. Synthesis of PEI-coated TiO_2_ nanoparticles (PEI-TiO_2_ NPs)

There are several synthesis methods available for tailoring TiO_2_ NPs, such as solvothermal, hydrothermal, sol-gel, chemical vapor deposition, atomic layer deposition, microemulsion, and reverse emulsion methods [28]. Among these, hydrothermal processes are particularly advantageous, as they can produce an entire anatase phase in the nanosize range and uniform distribution with high dispersion in polar or nonpolar solvents [29]. Additionally, this process is environmentally friendly, with low energy consumption and cost. In this study a hydrothermal method was utilized to produce TiO_2_ following the previously described procedure [29]. Briefly, titanium(IV) isopropoxide, Ti{OCH(CH_3_)_2_}_4_, was dissolved in n-propanol in a Teflon beaker. To this mixture, a combination of n-propanol and hydrochloric acid was slowly added. Following this, a mixture of distilled H_2_O and n-propanol was added to the same solution and stirred for 30 min. The prepared solution was then transferred to a hydrothermal reactor and heated at 150 °C for 2 h. The ratios of H_2_O/Ti{OCH(CH_3_)_2_}_4_ and HCl/Ti{OCH(CH_3_)_2_}_4_ were 2 and 0.2, respectively. The resultant NPs were isolated using an ultracentrifuge at 10,000 rpm and dried in a vacuum oven at 30 °C for 4 h. Subsequently, the TiO_2_ NPs were modified using the PEI polymer in the following procedure: TiO_2_ NPs (150 mg) were dispersed in 80 mL of distilled H_2_O and 2 g of branched PEI dissolved in 20 mL of deionized water was added to this solution. The pH of the mixture was adjusted to 10 by adding 0.1 M NaOH and heated to 50 °C for 2 h. After the reaction reached room temperature, the PEI-TiO_2_ NPs were separated by ultracentrifugation at 10,000 rpm for 15 min and washed with distilled H_2_O to remove the excess PEI. Finally, the PEI-TiO_2_ NPs were dried in a vacuum oven at 40 °C.

### 2.4. Synthesis of 2,6-diI_2_-BODIPY-linked TiO_2_ NPs (Bod-TiO_2_)

In a 50-mL round-bottom flask, PEI-modified TiO_2_ NPs (50 mg) were dispersed in MeOH:THF (1:1) (100 mL) under a nitrogen gas atmosphere. To this PEI-TiO_2_ solution, 2,6-diI_2_-BODIPY (30 mg), DCC (4 mg), and DMAP (2 mg) were added. The reaction mixture was stirred at room temperature overnight. The resulting 2,6-diI_2_-BODIPY-linked TiO_2_ NPs were collected using an ultracentrifuge at 10,000 rpm for 15 min, washed three times with a mixture of ethanol:water (1:1; 10 mL each time), and then dried in a vacuum oven at 40 °C.

### 2.5. Determination of the loading capacity of Bod PS onto TiO_2_ NPs

After the formation of Bod-TiO_2_ NPs, the NPs were separated from the reaction medium using an ultracentrifuge. The supernatant solution was purified using a silica gel flash column with a CHCl_3_:MeOH (3%) eluent system to remove excess DCC and DMAP reagent. Subsequently, the purified Bod PS was dissolved in DCM.

The loading efficiency (LE%) and loading capacity (LC%) of Bod onto TiO_2_ NPs was determined using a UV-Vis spectrometer [16]. Initially, a calibration curve was established using various concentrations of Bod (2 × 10^−5^, 1 × 10^−5^, 5 × 10^−6^, 2.5 × 10^−6^, and 1.25×10^−6^ M) in DCM (Supporting Information, Figure S8). Following the binding of Bod to the TiO_2_ NPs, the supernatant solution was diluted and its concentration was assessed using a UV-Vis spectrometer. The unknown Bod PS concentration was calculated using the Lambert–Beer equation of *A = ɛ*b.c where *A*, ɛ, b and *c* are the absorption, absorption coefficient (M^−1^cm^−1^), length of the light’s path (cm), and concentration of BODIPY (M), respectively. This analytical approach enabled the precise determination of the LE% and LC% of Bod according to Eqs. (1) and (2).


(1) $$LE\;(\%)=\frac{Total\;amount\;of\;PS\;(mg)-amount\;of\;PS\;in\;supernatant\;(mg)}{Total\;amount\;of\;PS\;(mg)}\times 100$$

(2) $$LC\;(\%)=\frac{Total\;amount\;of\;PS\;(mg)-amount\;of\;PS\;in\;supernatant\;(mg)}{Weight\;of\;the\;nanocarrier\;(mg)}\times 100$$

### 2.6. Photocatalytic degradation of bare PEI-TiO_2_ NPs and Bod-TiO_2_ NPs

The photocatalytic performance of the bare PEI-TiO_2_ NPs was evaluated through the degradation of methyl blue (MB) [30,31]. To conduct the photocatalytic degradation of MB by TiO_2_, an aqueous dispersion of PEI-coated TiO_2_ NPs (PEI-TiO_2_ NPs) (0.5 wt.%) was first prepared by subjecting them to ultrasonication for 15 min at room temperature. To the MB (10 ppm, 20 mL) solution, PEI-TiO_2_ NP solution (0.5 mL) was introduced and stirred for 1 h for adsorption–desorption equilibrium in the dark. The mixture was then exposed to UV light for a determined time interval, and the MB concentration was measured every 5 min for 25 min using a UV-Vis spectrometer (PerkinElmer Lambda 650). The same procedure was repeated for testing the photocatalytic behavior of Bod-TiO_2_. To evaluate the stability of MB under UV radiation in the absence of a photocatalyst, a blank experiment was conducted. MB was exposed to UV radiation for an extended period of time, and the intensity of the absorbance was not changed at 655 nm, the MB’s maximum absorption wavelength. In this experiment, MB was chosen as a dye due to its high affinity for metal oxide surfaces, clear optical absorption, and resistance to light degradation. The degradation efficiency of MB, represented as D%, was calculated using Eq. (3).


(3) $$D\;(\%)=\frac{C_{o}-C_{t}}{C_{o}}\times 100$$

Here, *C_0_* and *C_t_* represent the concentrations of MB at time 0 and *t* (min), respectively.

### 2.7. Singlet oxygen generation using bare Bod-I_2_ and Bod-TiO_2_ NPs

As a singlet oxygen probe, DPBF was selected to study the ^1^O_2_ generation and its absorbance was adjusted to approximately 1.00 in air-saturated methanol [10,11,17]. The Bod PS (1 mg/mL methanol) was added to DPBF in methanol (2 mL) within a UV-quartz cuvette. Afterward, the mixture was exposed to UV light at 532 nm every 30 min for 150 s using the UV-Vis spectrometer. The reduction in the absorbance intensity of DPBF at 410 nm was recorded to determine the ^1^O_2_ generation ability. The same procedure was repeated for the Bod-TiO_2_ NPs. However, the dispersion of Bod-TiO_2_ was achieved using 1 mg/mL in methanol:distilled H_2_O (1:1). The exposure time to UV light was adjusted to every 10 min for a total of 150 min. The measurements were performed three times and the average values were reported.

## 3. Results and discussion

### 3.1. Physical and chemical characteristics of TiO_2_ NPs

**Figure 2:**
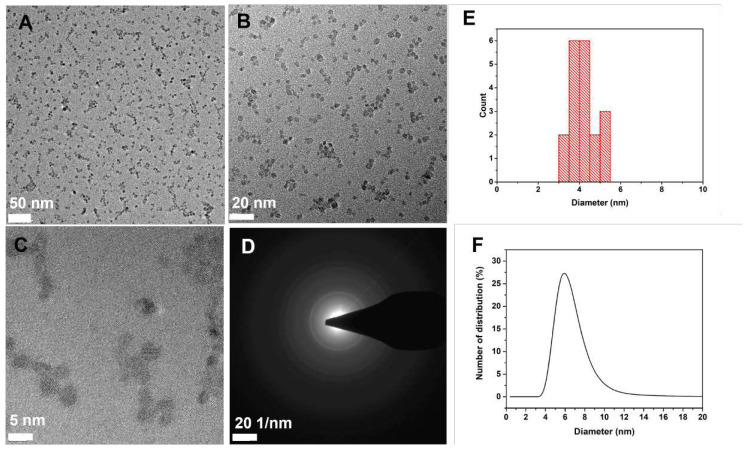
TEM images of the TiO_2_ NPs (**A**–**C**), selected-area electron diffraction pattern (**D**), size distribution calculated using ImageJ (**E**), and hydrodynamic size distribution obtained by DLS (**F**).

In this study, an acid-catalyzed hydrothermal method was employed to synthesize TiO_2_ NPs as evidenced by TEM images (Figures 2a–2c). The selected-area electron diffraction results by TEM confirmed the high crystallinity of the TiO_2_ NPs (Figure 2d), which were obtained with a pure anatase crystal phase, as analyzed by X-ray diffraction (XRD) technique (Supporting Information, Figure S9). Based on the TEM images, the NPs exhibited a diameter of 4 ± 2 nm and displayed a spherical morphology (Figure 2e). In an aqueous solution, the diameter was measured to be 6.4 nm with a polydispersity index (PdI) value of 0.16, as determined by DLS (Figure 2f). The low PdI value indicated monodispersity in distilled H_2_O.

**Figure 3:**
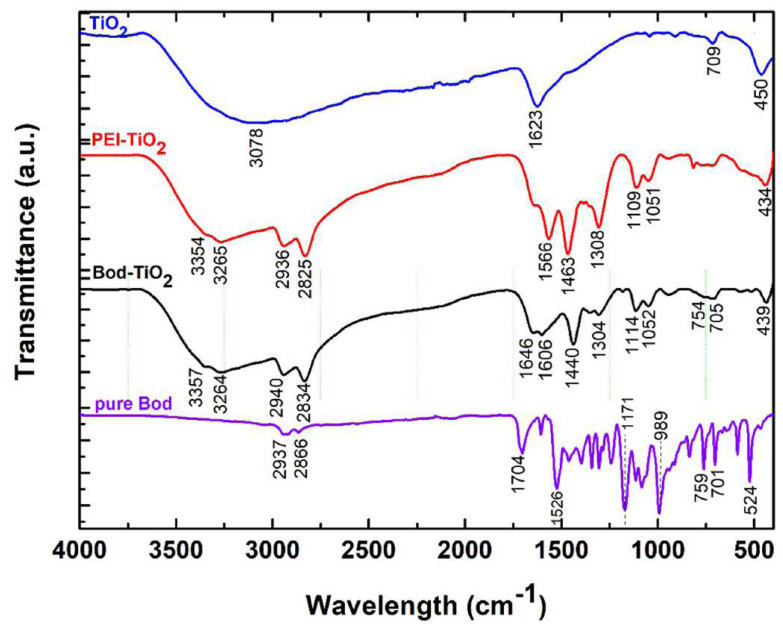
FTIR spectra of the synthesized TiO_2_, PEI-TiO_2_, Bod PS, and Bod-TiO_2_ NPs.

The covalent conjugation between Bod6-COOH and TiO_2_-NH_2_ species was achieved using FTIR (Figure 3). The bending vibrations of the Ti-O-Ti bonds in the TiO_2_ NPs were responsible for the broad peak observed at 450 cm^−1^ in Figure 3. The broad peaks at 3078 cm^−1^ and 1623 cm^−1^ were attributed to stretching and bending vibrations of the hydroxyl groups, respectively, on the TiO_2_ surface [32]. After coating the TiO_2_ surface with PEI, the peaks of the amine group appeared [33]. The peaks at 3354 and 3265 cm^−1^ and at 2936 and 2825 cm^−1^ were attributed to the stretching vibrations of the N-H bond and the aliphatic C-H bond of the PEI polymer, respectively. The peaks at 1566, 1463, and 1306 cm^−1^ correspond to the out-of-plane bending of the N-H bonding and aliphatic C-H in-plane bending of CH_2_ and CH_3_, respectively. Additionally, the vibrations at 1109–1051 cm^−1^ were related to the in-plane bending of N-H. On the other hand, the pure BODIPY PS exhibited characteristics peaks at 1526 cm^−1^ due to B-F vibration. The stretching vibration peak of the carbonyl group (C=O) in the Bod6 appears at 1704 cm^−1^. After the conjugation of Bod6 with TiO_2_ NPs, three characteristic amide peaks emerged at 1646 cm^−1^ (first amide bond, stretching of C=O bond), 1602 (second amide bond, stretching of N-H), and 1440 cm^−1^ (third amide bond, stretching of C-N).

### 3.2. Photophysical characterizations of the Bod PS and Bod-TiO_2_ NPs

**Figure 4:**
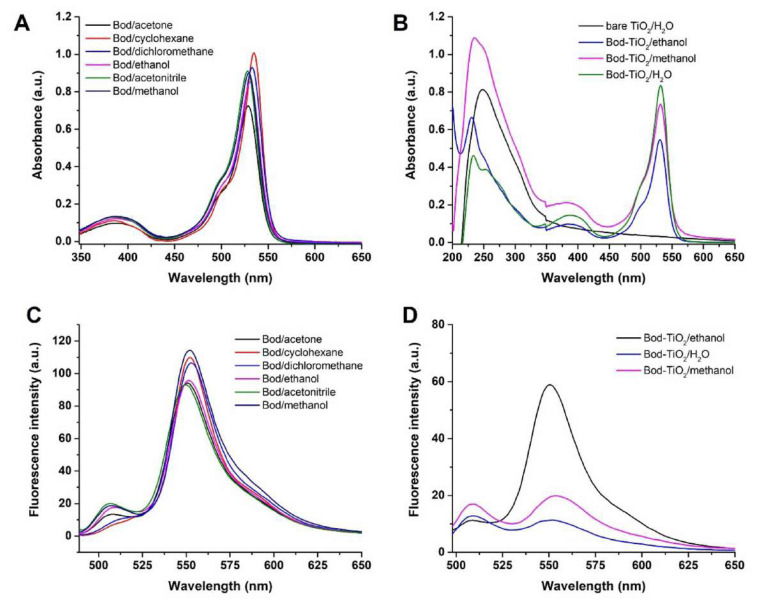
Absorbance of Bod6 (**A**) and Bod6-TiO_2_ (**B**), and fluorescence emissions of Bod6 (**C**) and Bod-TiO_2_ (**D**).

The absorption and emission maxima of the Bod PS were ascertained by means of UV-Vis and fluorescence spectrometry (Agilent Cary Eclipse) in various solvent systems (Figures 4a–4d). The solvents were chosen based on their dielectric constants. As clearly indicated in Table 1, an increase in the dielectric constant of the solvent led to an increase in the Stoke shift.

After binding Bod PS to TiO_2_, the absorption and emission wavelengths of Bod PS remained unchanged compared to bare Bod PS in both ethanol and methanol solvents (Table 2). However, covalent conjugation with Bod6 led to a blue shift, with the λ_max_ of TiO_2_ appearing at 230 nm, 233 nm, and 235 nm in ethanol, water, and methanol, respectively, whereas bare TiO_2_ exhibited an absorption maximum at 247 nm (Figure 4b) [34]. This observation suggested that Bod PS covered the surface of TiO_2_, resulting in a hypsochromic shift in the UV region.

**Figure 5:**
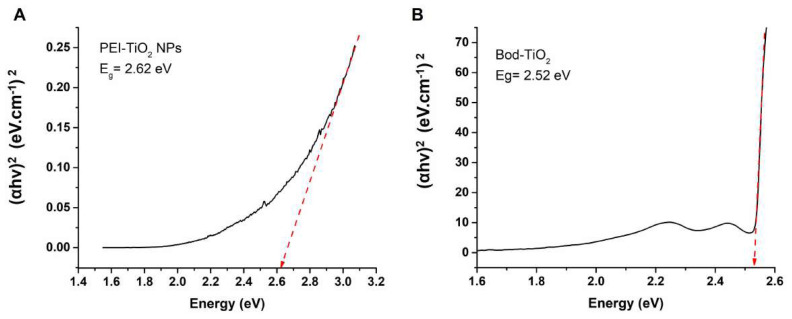
Band gap energy (*E_g_*) calculations using Tauc plots. A linear part of the plot was extrapolated to the x-axis for PEI-TiO_2_ (**A**) and Bod-TiO_2_ (**B**).

The conjugation of the Bod scaffold with PEI-TiO_2_ also impacted the energy band gap of TiO_2_. The band gap energy of the PEI-coated TiO_2_ NP, both unmodified and modified with Bod PS, was determined using the Tauc plot method based on the energy of incident photons. The respective values were found to be 2.62 eV and 2.52 eV (Figures 5a and 5b). The functionalization of the TiO_2_ surface with BODIPY molecules resulted in a slight decrease in the band gap of TiO_2_. This decrease was attributed to the low band gap energy of the BODIPY molecules [35,36].

### 3.3. Loading efficiency and loading capacity of TiO_2_ NPs

The quantification of Bod adsorption onto the surface of TiO_2_ is of paramount significance as it directly impacts the efficiency of singlet oxygen production. Therefore, two parameters become important in evaluating the PS contents of the NPs: the loading efficiency (LE%) and loading capacity (LC%). LE% denotes the proportion of PS that has been effectively enclosed within the NP [16]. On the other hand, LC% refers to the quantity of PS incorporated per unit weight of the NP. In this study, the values of LE% and LC% were calculated and were found to be 90.8% and 54.5%, respectively, which showed that most of the PS had been bonded to the TiO_2_ NPs.

### 3.4. Investigation of photocatalytic behavior

**Figure 6:**
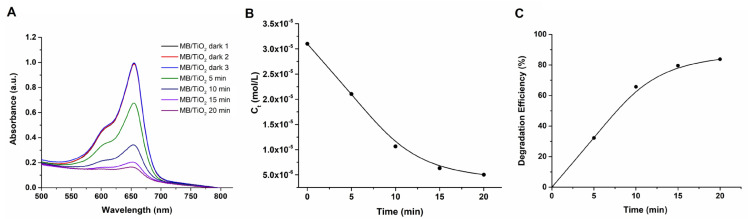
Change of absorbance of MB by TiO_2_ photocatalyst (**A**), concentration of MB vs. time (**B**), and photocatalytic degradation efficiency % of MB with time (**C**).

To investigate the photocatalytic characteristics of the TiO_2_ NPs, an examination of the degradation of MB by TiO_2_ NPs was conducted over time under UV illumination, as illustrated in Figure 6a. The changes in MB concentrations with respect to time are presented in Figure 6b. Accordingly, it was ascertained that the TiO_2_ NPs displayed commendable photocatalytic efficacy, achieving 83.8% degradation efficiency for the MB dye within a 20-min interval as depicted in Figure 6c.

Furthermore, the time-dependent degradation of MB by TiO_2_ under UV light was analyzed kinetically by applying pseudo-first-order (PFO) and pseudo-second-order (PSO) kinetic models, as formulated in Eqs. (4) and (5), respectively [37,38].


(4) $$In\;\left(\frac{C_{0}}{C_{t}}\right)=k_{1}.t$$

(5) $$\frac{1}{C_{t}}=\frac{1}{C_{0}}+k_{2}.t$$

**Figure 7:**
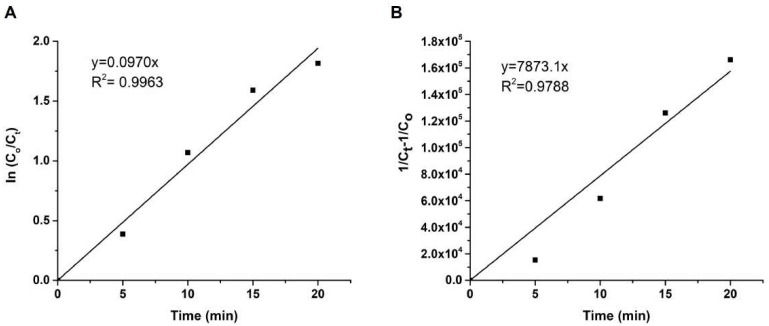
Pseudo-first-order (**A**) and pseudo-second-order (**B**) kinetic models for the degradation of MB.

Here, *C_0_* (mg/L) is the initial concentration of MB and *C_t_* represents the concentration of MB after the initiation of the photocatalytic process at specific time intervals denoted as *t* (min). Meanwhile, *k_1_* (1/min) and *k_2_* (L/mol min) correspond to the rate constants of the PFO and PSO kinetic models, respectively. Figures 7a and 7b depict the fitting of the kinetic data acquired for the degradation of the MB dye by TiO_2_ NPs to both the PFO and PSO kinetic models. The experimental findings revealed that the correlation coefficient for the PFO kinetic model (R^2^ = 0.9963) is greater than that of the PSO kinetic model (R^2^ = 0.9788), indicating that the photocatalytic process follows the PFO kinetic model with *k_1_* = 0.097 min^−1^ for the simultaneous elimination of MB cationic dyes by TiO_2_ NPs.

**Figure 8:**
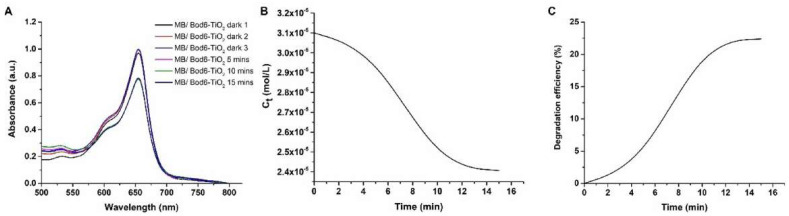
Change of absorbance of MB by Bod-TiO_2_ photocatalyst (**A**), concentration of MB vs. time (**B**), and photocatalytic degradation efficiency % of MB with time (**C**).

The photocatalytic activity of Bod-TiO_2_ NPs was also determined by examining the time-dependent degradation of MB dye by Bod-TiO_2_ NPs under UV light. An evident reduction in the absorbance of MB was noted within a span of 15 min, as illustrated in Figure 8a. Accordingly, a time-dependent change in the concentration of MB is graphically represented in Figure 8b. However, upon binding the Bod PS to the TiO_2_ NPs, a linear degradation similar to that of bare TiO_2_ NPs was not observed (Figure 8c). Nevertheless, a decomposition of 22.4% was noted within a span of 15 min (Figure 8c). This implies that the Bod PS was coated on the surface of the TiO_2_ NPs, resulting in the absorption of most of the light by the Bod PS. Thus, the catalytic efficiency of TiO_2_ was decreased compared to unmodified TiO_2_ NPs.

### 3.5. Singlet oxygen generation

**Figure 9:**
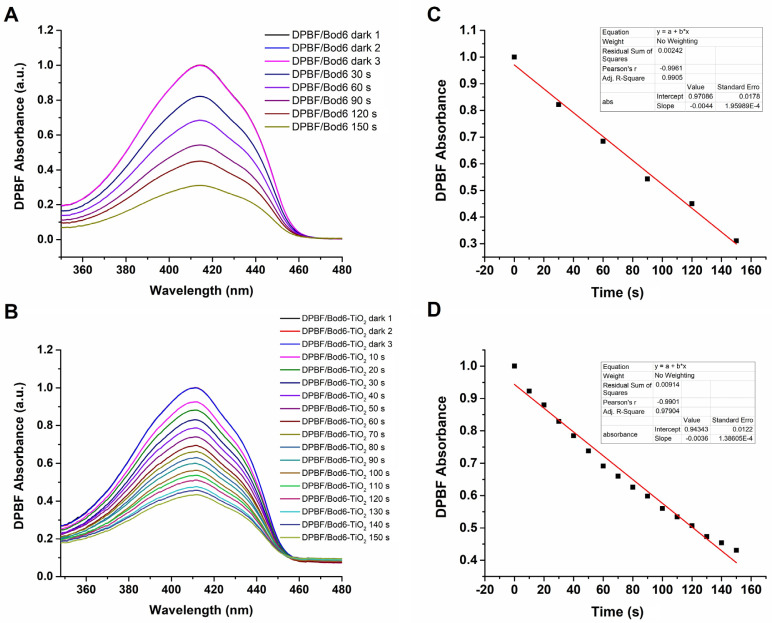
Time-dependent photodegradation of Bod (**A**) and Bod-TiO_2_ NPS (**B**); rate curves of the DPBF photodegradation of Bod (**C**) and Bod-TiO_2_ NPS (**D**) at 410 nm.

To evaluate the singlet oxygen generation capacity of Bod and Bod-TiO_2_, the ^1^O_2_ probe known as DPBF was employed as an indicator. The experiment was conducted in air-saturated methanol under light of 532 nm. As depicted in Figure 9, the absorbance of DPBF at 410 nm gradually declined for both Bod and Bod-TiO_2_ over a duration of 150 min. The Bod-TiO_2_ NPs displayed a 56.9 ± 1.13% reduction in DPBF absorbance, while pure Bod PS demonstrated a more pronounced decrease of 68.9 ± 0.06% in 150 min (Figures 9a and 9b). In Figures 9c and 9d, a linear decrease is demonstrated, resulting from the reduction in absorbance observed in Figures 9a and 9b. Accordingly, for every 30 s of irradiation with LED light of 532 nm, the Bod PS demonstrated a reduction of 20.6 ± 2.04% in DPBF absorbance, while Bod-TiO_2_ exhibited a slightly lower decrease of 15.5 ± 1.10%. A comparative graph illustrating the decrease in absorbance together with standard deviation values (n = 3) is provided in the Supporting Information (Figure S10). However, the difference between the PSs in terms of singlet oxygen production on average was only 11.5% in total, suggesting that the Bod-TiO_2_ NPs maintained a comparable level of efficacy, which also supports the high content of Bod bonded to the TiO_2_ NPs. This highlights the robustness of the Bod-TiO_2_ NPs in maintaining high singlet oxygen production efficiency. In the control experiments, no change in absorbance intensity was observed in the absence of light for the pure Bod PS or Bod-TiO_2_. When DPBF and bare TiO_2_ were subjected to LED light of 532 nm, no alterations were observed (Supporting Information, Figure S11).

## 4. Conclusion

Despite the nonsoluble nature of Bod PS, stable dispersion was achieved in physiological media over a week after integrating the Bod with TiO_2_ NPs. Upon the binding of Bod, the absorption maxima of TiO_2_ shifted to a range of 230–235 nm due to the high coverage of Bod on the surface, resulting in light absorption. This was also supported by the yields of LE% and LC%, which were 90.8% and 54.5%, respectively. Photocatalytic behavior was evaluated using MB degradation under UV light and the results demonstrated 83.8% photodegradation efficiency. The kinetics fit a PFO kinetic model with *k_1_* = 0.097 min^−1^. However, after binding with Bod PS, the photocatalytic activity decreased to 22.4%. Nevertheless, the Bod-TiO_2_ NPs exhibited a singlet oxygen generation rate of 56.9 ± 1.13%, which could be considered moderate efficiency in terms of PDT activity. As a result, the Bod-TiO_2_ NPs display higher dispersibility in aqueous solutions, have moderate photocatalytic activity, and hold potential for PDT applications.

## Supporting Information

^1^H NMR and ^13^C NMR spectra of compounds **3**, **5**, and **6**

Figure S1 ^1^H NMR spectrum of 6-(4-formylphenoxy)hexanoic acid (**3**) (CDCl_3_, 400 MHz).

Figure S2 ^13^C NMR spectrum of 6-(4-formylphenoxy)hexanoic acid (**3**) (CDCl_3_, 100 MHz).

Figure S3 ^1^H NMR spectrum of 1,3,5,7-tetramethyl-8-(4-(5-carboxypentyloxy)phenyl-4,4-difloro-4-bora-3a,4a-diaza-s-indacene (**5**) (CDCl_3_, 400 MHz).

Figure S4 ^13^C NMR spectrum of 1,3,5,7-tetramethyl-8-(4-(5-carboxypentyloxy)phenyl-4,4-difloro-4-bora-3a,4a-diaza-s-indacene (**5**) (CDCl_3_, 100 MHz).

Figure S5 ^1^H NMR spectrum of 1,3,5,7-tetramethyl-2,6-diiyodo-8-(4-(5-carboxypentyloxy)phenyl-4,4-difloro-4-bora-3a,4a-diaza-s-indacene (**6**) (CDCl_3_, 400 MHz).

Figure S6 ^13^C NMR spectrum of 1,3,5,7-tetramethyl-2,6-diiyodo-8-(4-(5-carboxypentyloxy)phenyl-4,4-difloro-4-bora-3a,4a-diaza-s-indacene (**6**) (CDCl_3_, 100 MHz).

Figure S7 ESI-HRMS spectrum of 1,3,5,7-tetramethyl-2,6-diiyodo-8-(4-(5-carboxypentyloxy)phenyl-4,4-difloro-4-bora-3a,4a-diaza-s-indacene (**6**).

Figure S8 UV-Vis spectrum of 2 × 10^−5^, 1 × 10^−5^, 5 × 10^−6^, 2.5 × 10^−6^, and 1.25 × 10^−6^ M Bod solutions and the supernatant Bod solution in DCM (**A**); calibration plot of Bod (**B**).

Figure S9 XRD pattern of the synthesized TiO_2_ (black line) and reference anatase TiO_2_ NPs (JCPDS Card No: 78-24869).

Figure S10 Average decline in DPBF absorbance at 411 nm for Bod PS (**A**) and Bod-TiO_2_ (**B**) presented as mean values ± standard deviation; n = 3.

Figure S11 Control experiments: UV-Vis spectra of pure Bod, Bod-TiO_2_, bare TiO_2_, and DPBF.

## Figures and Tables

**Table 1:** Photophysical properties of Bod PS in different solvents.

Solvent	Dielectric constant (ɛ)	Absorption λ_abs(max)_, nm	Emission λ_em(max)_, nm	Stoke shift (cm^−1^)
Cyclohexane	2.02	535	552	576
Dichloromethane	8.93	533	553	679
Acetone	20.7	529	550	722
Ethanol	24.55	530	551	719
Methanol	32.70	529	552	788
Acetonitrile	37.50	528	549	724

**Table 2:** Absorption and emission wavelengths of Bod-TiO_2_ NPs in H_2_O, methanol, and ethanol.

	Solvents		

Samples	H_2_O	Methanol	Ethanol
	(ɛ: 80.40)	(ɛ: 32.70)	(ɛ: 24.55)

Bod-TiO_2_	TiO_2_ λ_abs_: 233	TiO_2_ λ_abs_: 235	TiO_2_ λ_abs_: 230
	Bod λ_abs_: 531; λ_em(max)_: 554 nm	Bod λ_abs_: 531; λ_em(max)_: 553 nm	Bod λ_abs_: 531; λ_em(max)_: 550 nm
