# Supplementary figures and images for: Exploring the photocatalytic and photodynamic effects of BODIPY-linked titanium dioxide nanoparticles
Source: Turk J Chem. 2023 Sep 30;47(6):1407–19. doi: 10.55730/1300-0527.3623 (PMC10965179; doi:10.55730/1300-0527.3623)

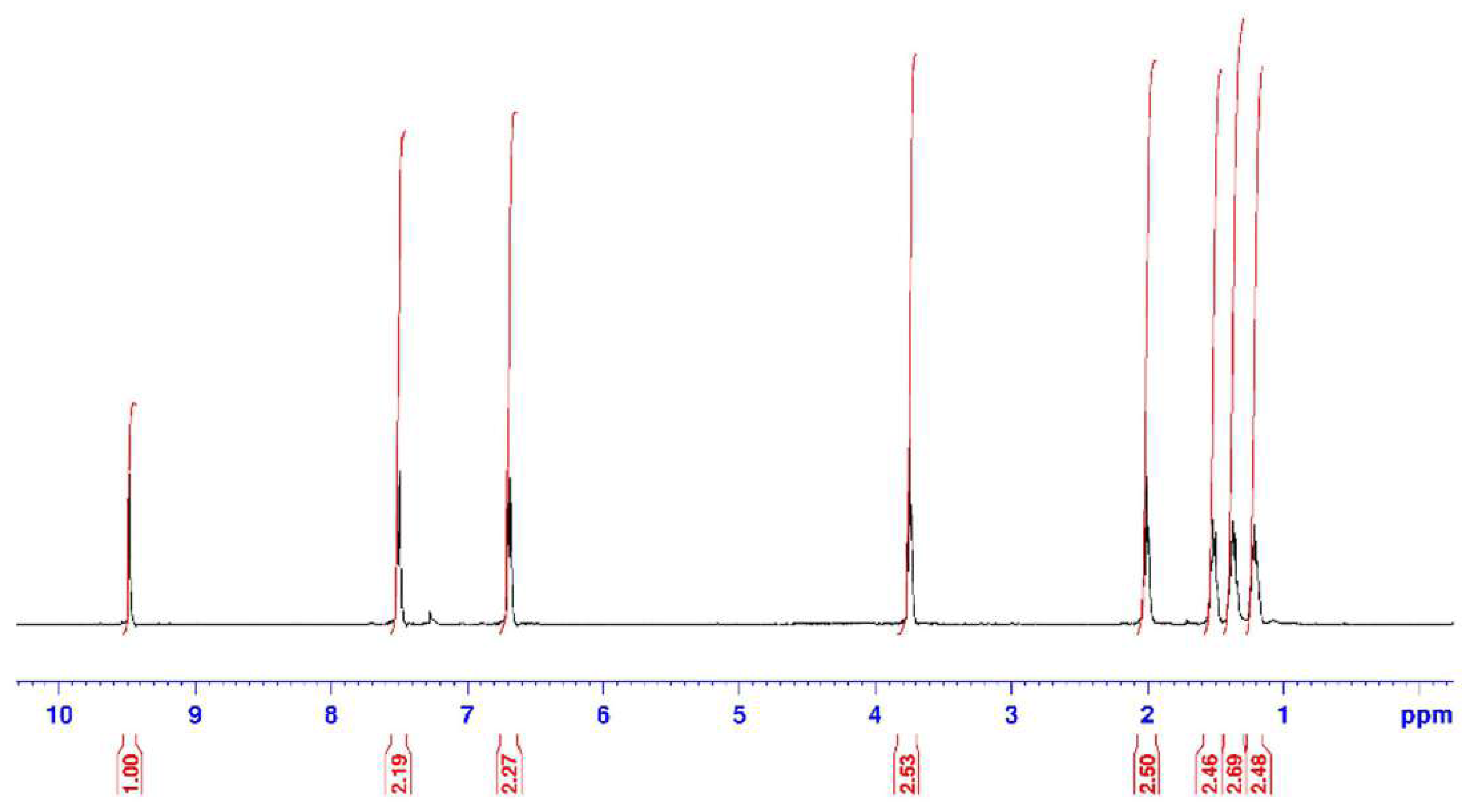

Supplement: Figure S1 — 1H NMR spectrum of 6-(4-formylphenoxy)hexanoic acid (3) (CDCl3, 400 MHz). [file tjc-47-06-1407s1.tif]

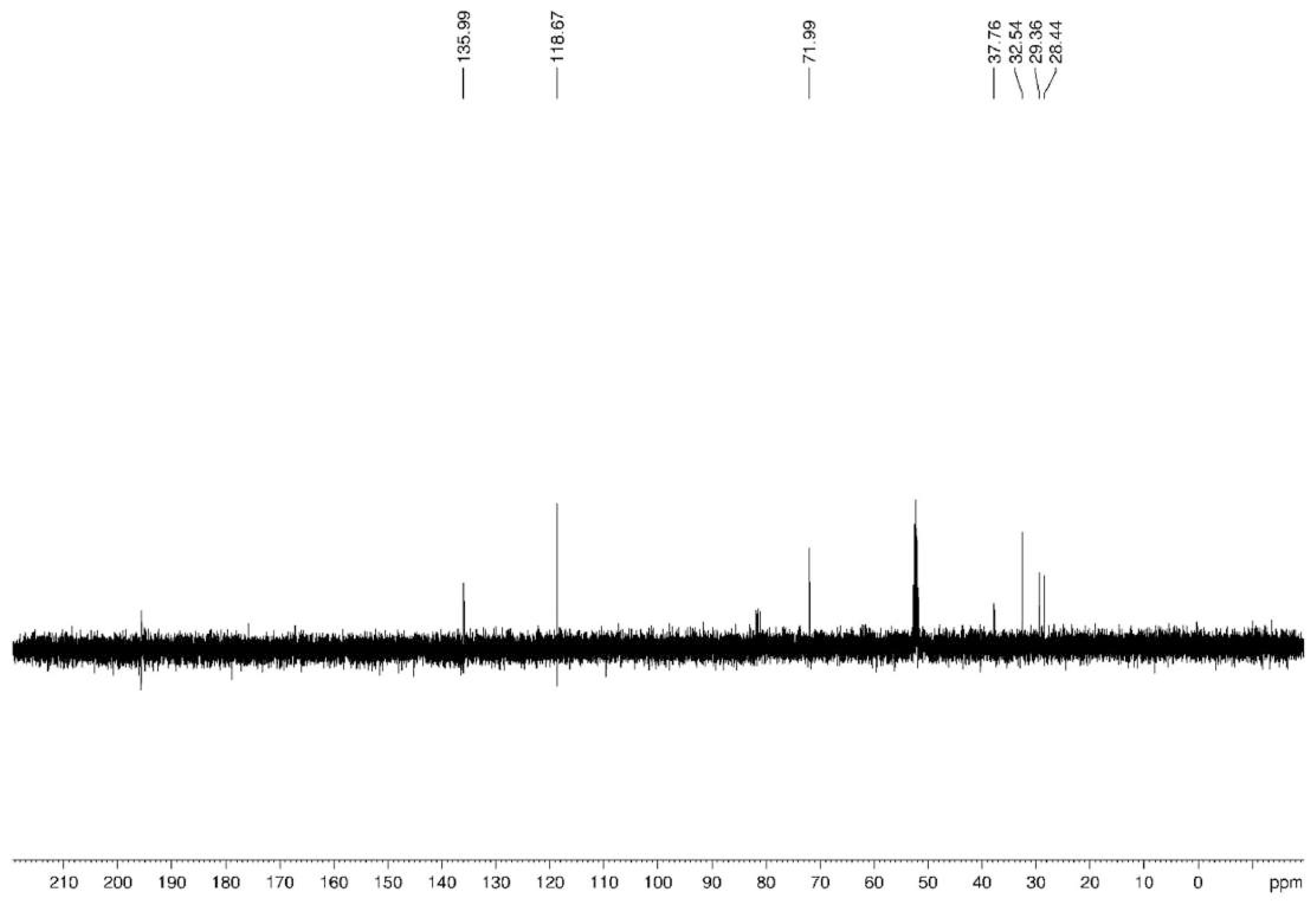

Supplement: Figure S2 — 13C NMR spectrum of 6-(4-formylphenoxy)hexanoic acid (3) (CDCl3, 100 MHz). [file tjc-47-06-1407s2.tif]

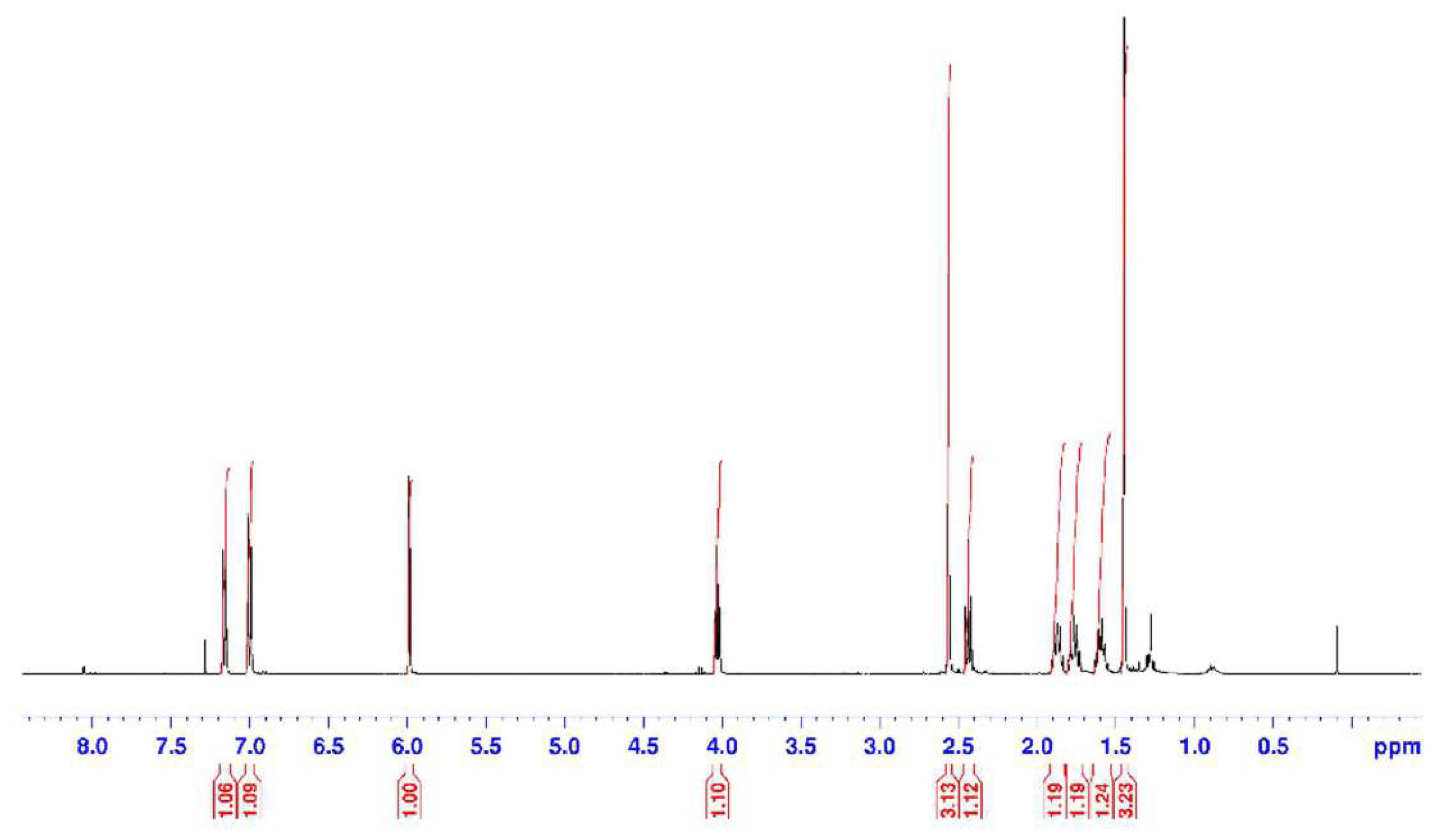

Supplement: Figure S3 — 1H NMR spectrum of 1,3,5,7-tetramethyl-8-(4-(5-carboxypentyloxy)phenyl-4,4-difloro-4-bora-3a,4a-diaza-s-indacene (5) (CDCl3, 400 MHz). [file tjc-47-06-1407s3.tif]

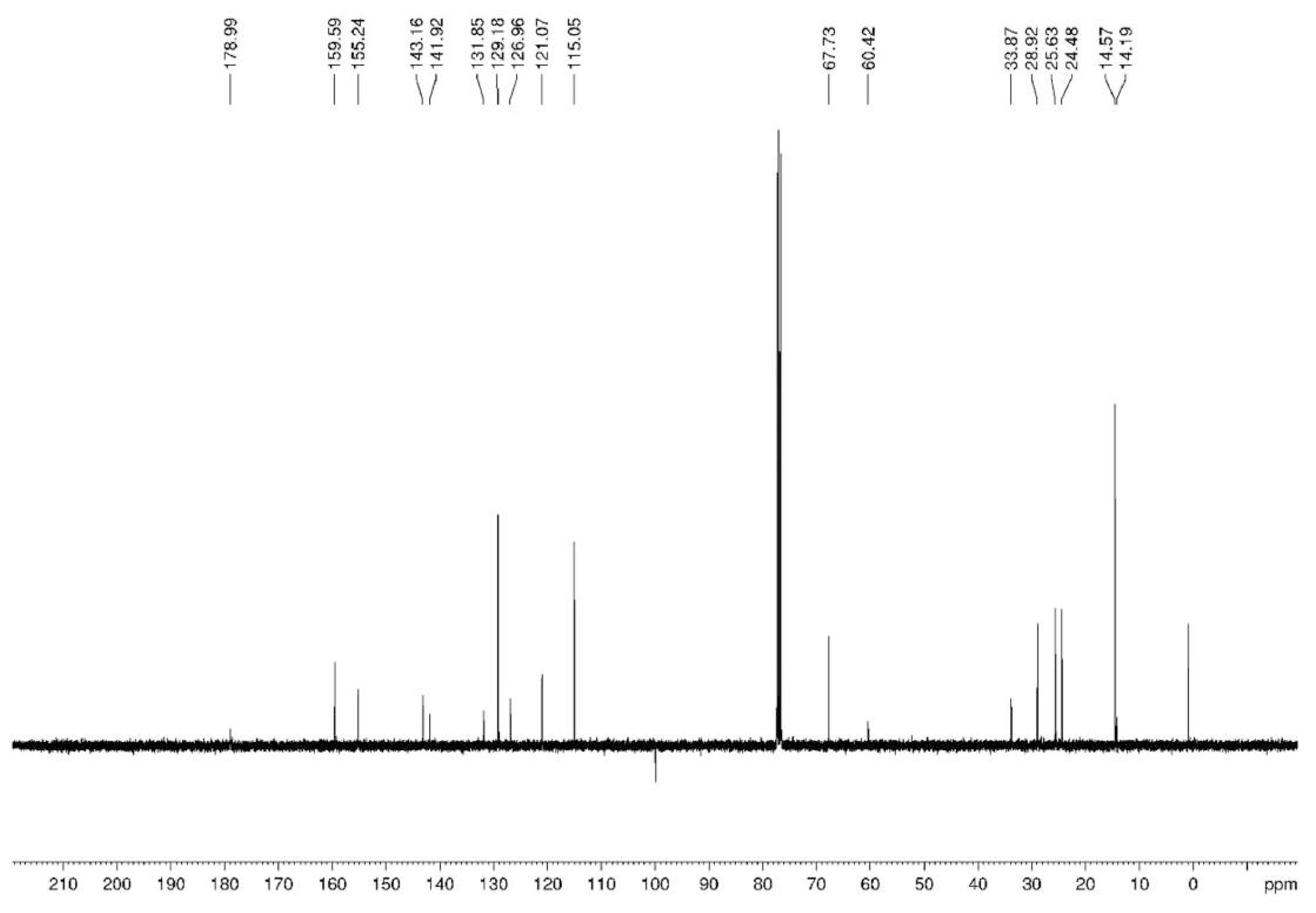

Supplement: Figure S4 — 13C NMR spectrum of 1,3,5,7-tetramethyl-8-(4-(5-carboxypentyloxy)phenyl-4,4-difloro-4-bora-3a,4a-diaza-s-indacene (5) (CDCl3, 100 MHz). [file tjc-47-06-1407s4.tif]

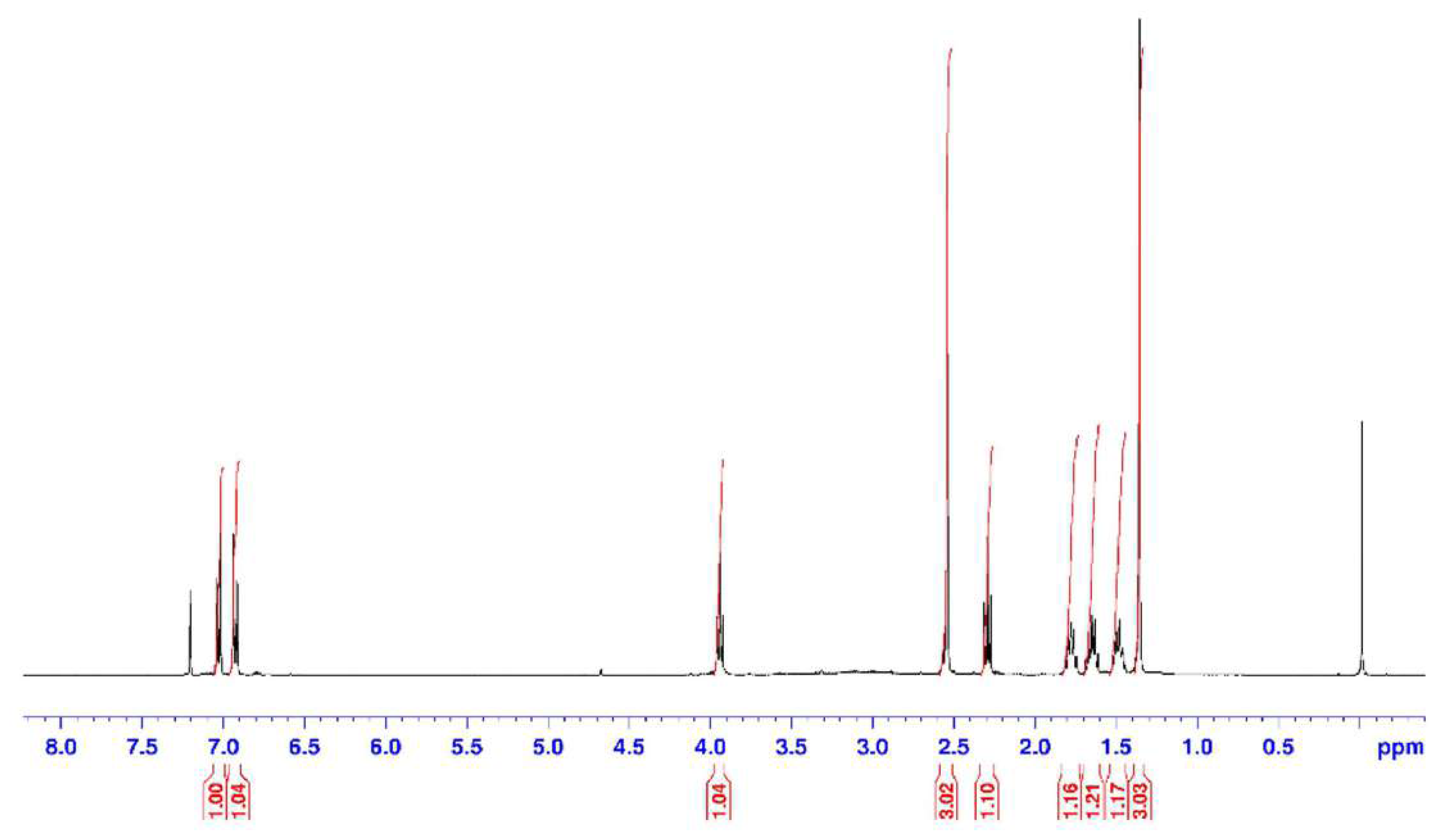

Supplement: Figure S5 — 1H NMR spectrum of 1,3,5,7-tetramethyl-2,6-diiyodo-8-(4-(5-carboxypentyloxy)phenyl-4,4-difloro-4-bora-3a,4a-diaza-s-indacene (6) (CDCl3, 400 MHz). [file tjc-47-06-1407s5.tif]

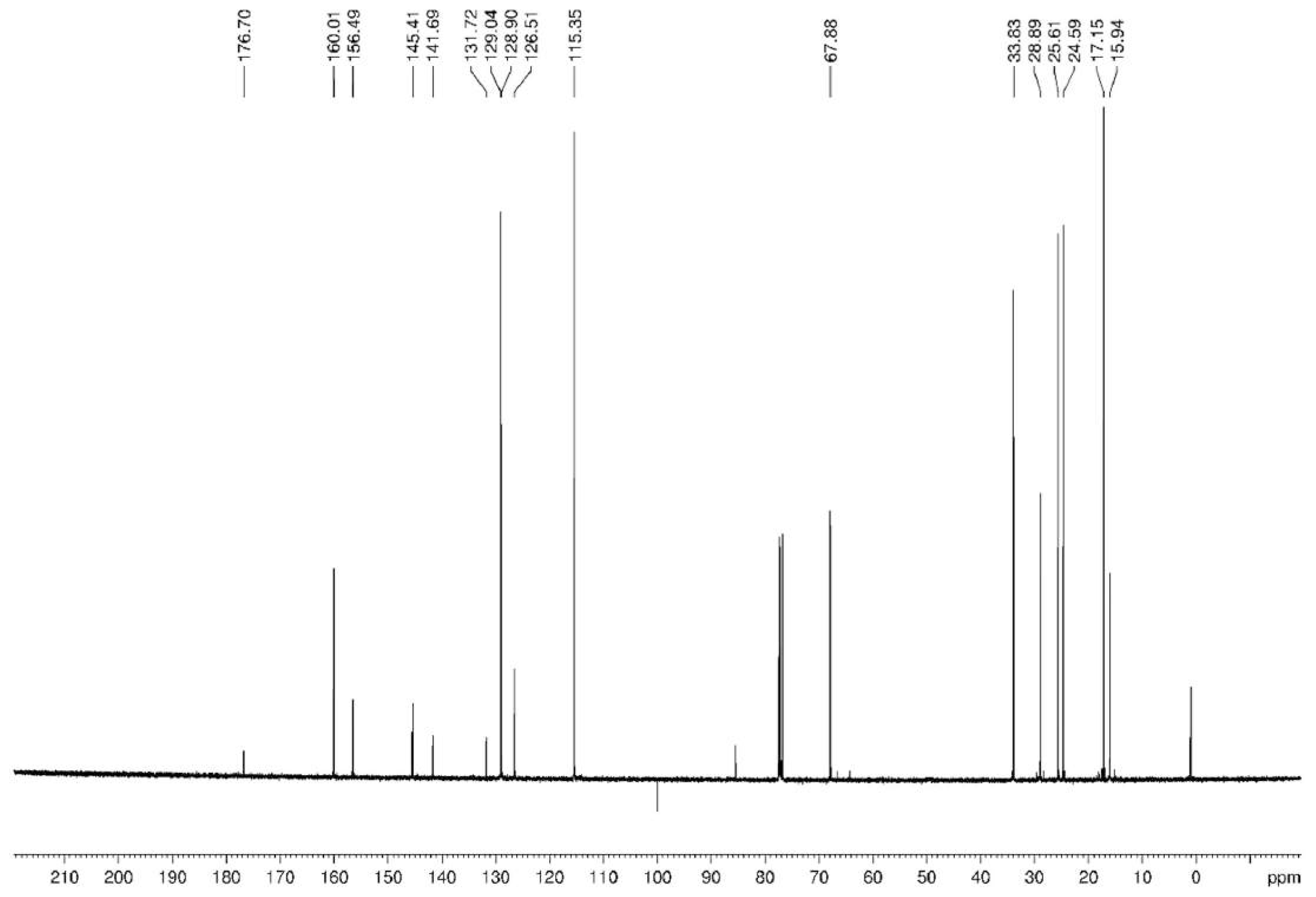

Supplement: Figure S6 — 13C NMR spectrum of 1,3,5,7-tetramethyl-2,6-diiyodo-8-(4-(5-carboxypentyloxy)phenyl-4,4-difloro-4-bora-3a,4a-diaza-s-indacene (6) (CDCl3, 100 MHz). [file tjc-47-06-1407s6.tif]

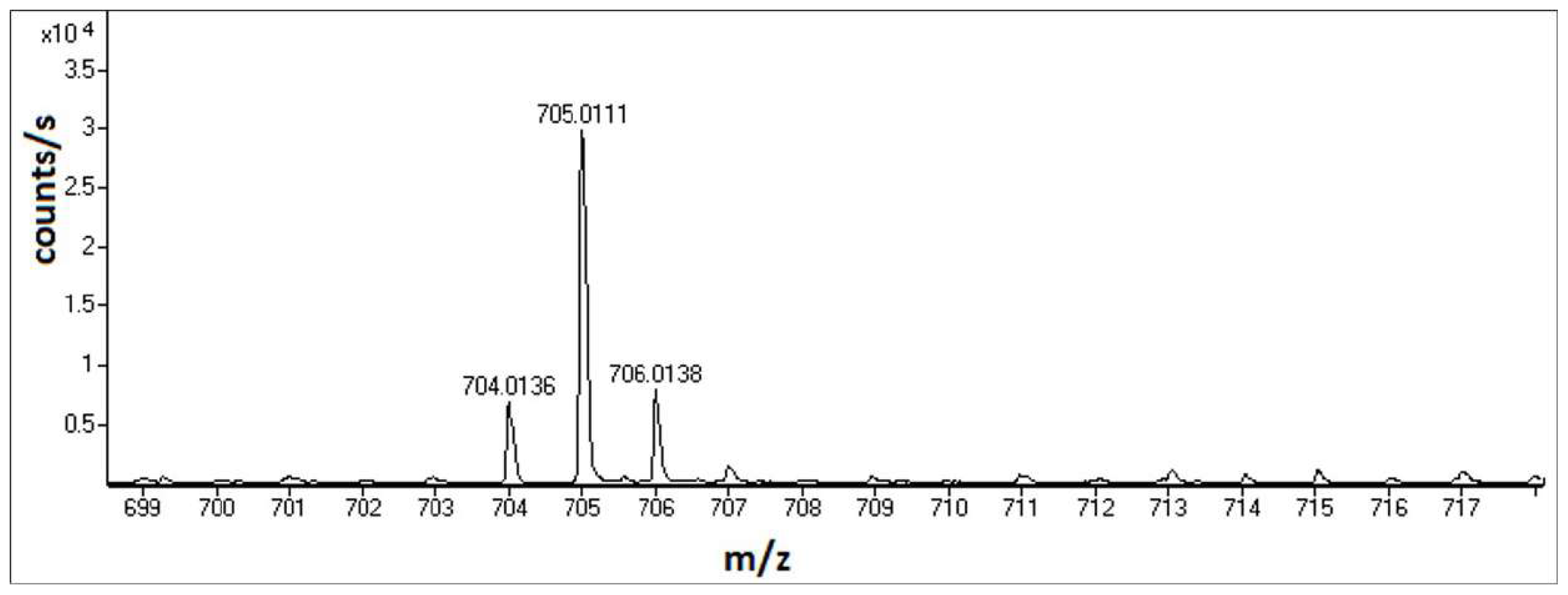

Supplement: Figure S7 — ESI-HRMS spectrum of 1,3,5,7-tetramethyl-2,6-diiyodo-8-(4-(5-carboxypentyloxy)phenyl-4,4-difloro-4-bora-3a,4a-diaza-s-indacene (6). [file tjc-47-06-1407s7.tif]

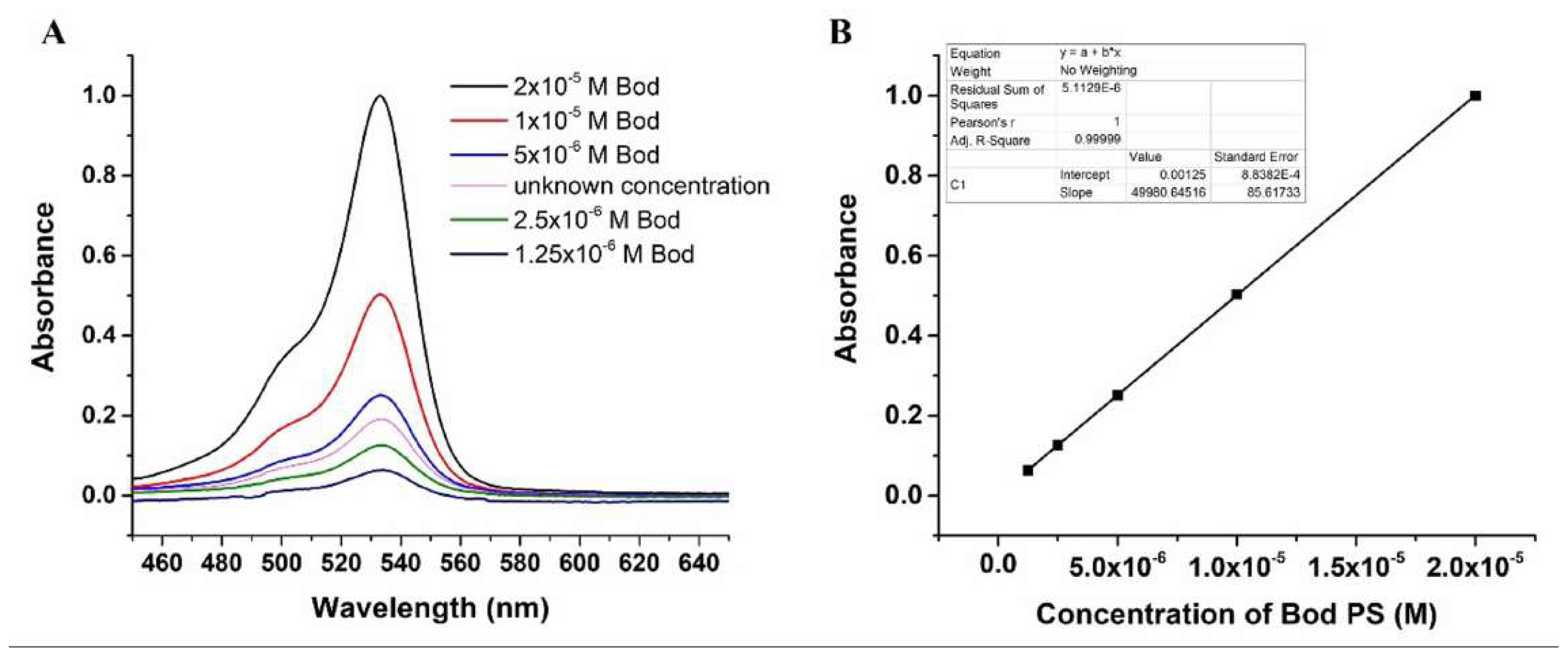

Supplement: Figure S8 — UV-Vis spectrum of 2 × 10^−5^, 1 × 10^−5^, 5 × 10^−6^, 2.5 × 10^−6^, and 1.25 × 10^−6^ M Bod solutions and the supernatant Bod solution in DCM (A); calibration plot of Bod (B). [file tjc-47-06-1407s8.tif]

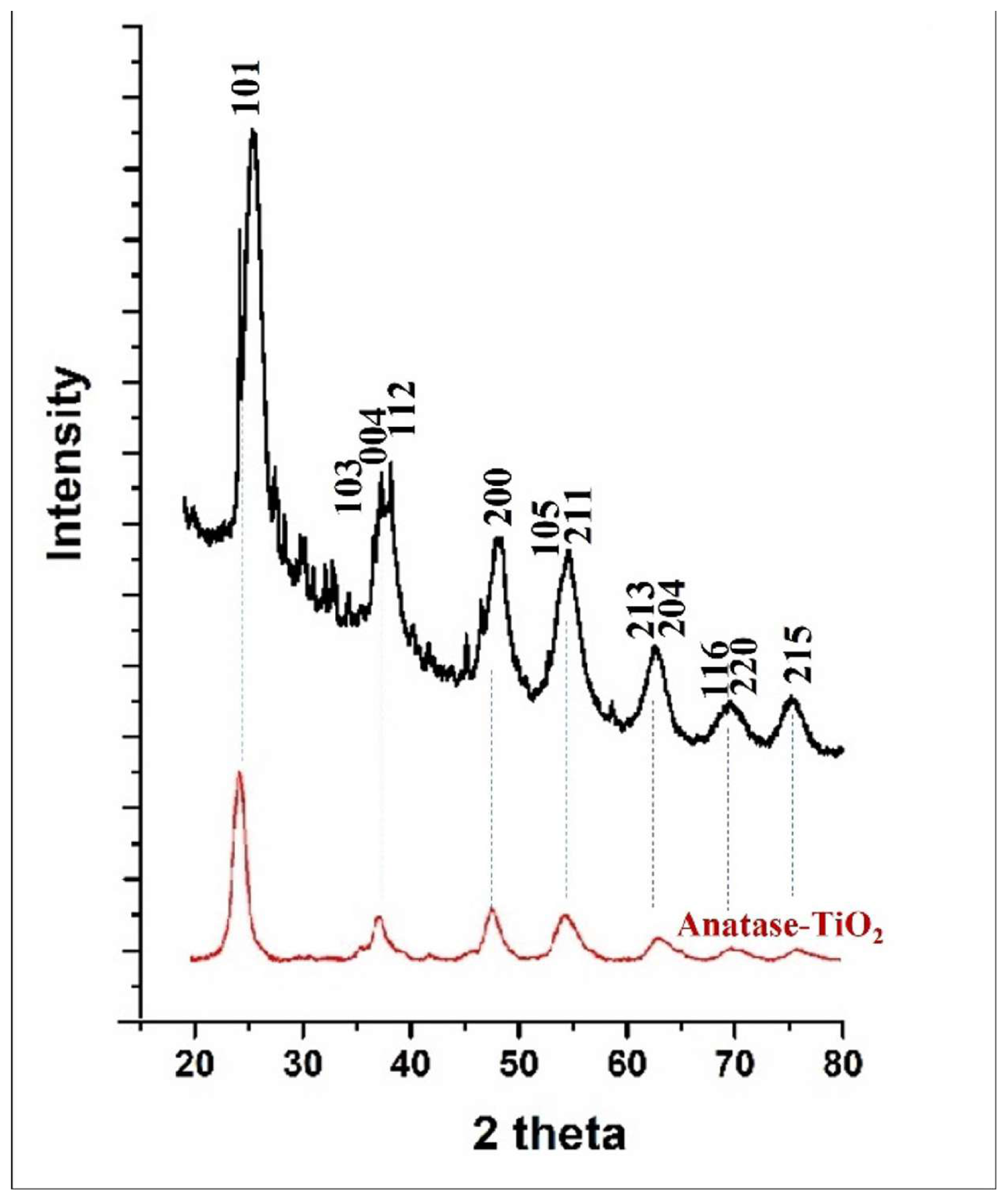

Supplement: Figure S9 — XRD pattern of the synthesized TiO2 (black line) and reference anatase TiO2 NPs (JCPDS Card No: 78-24869). [file tjc-47-06-1407s9.tif]

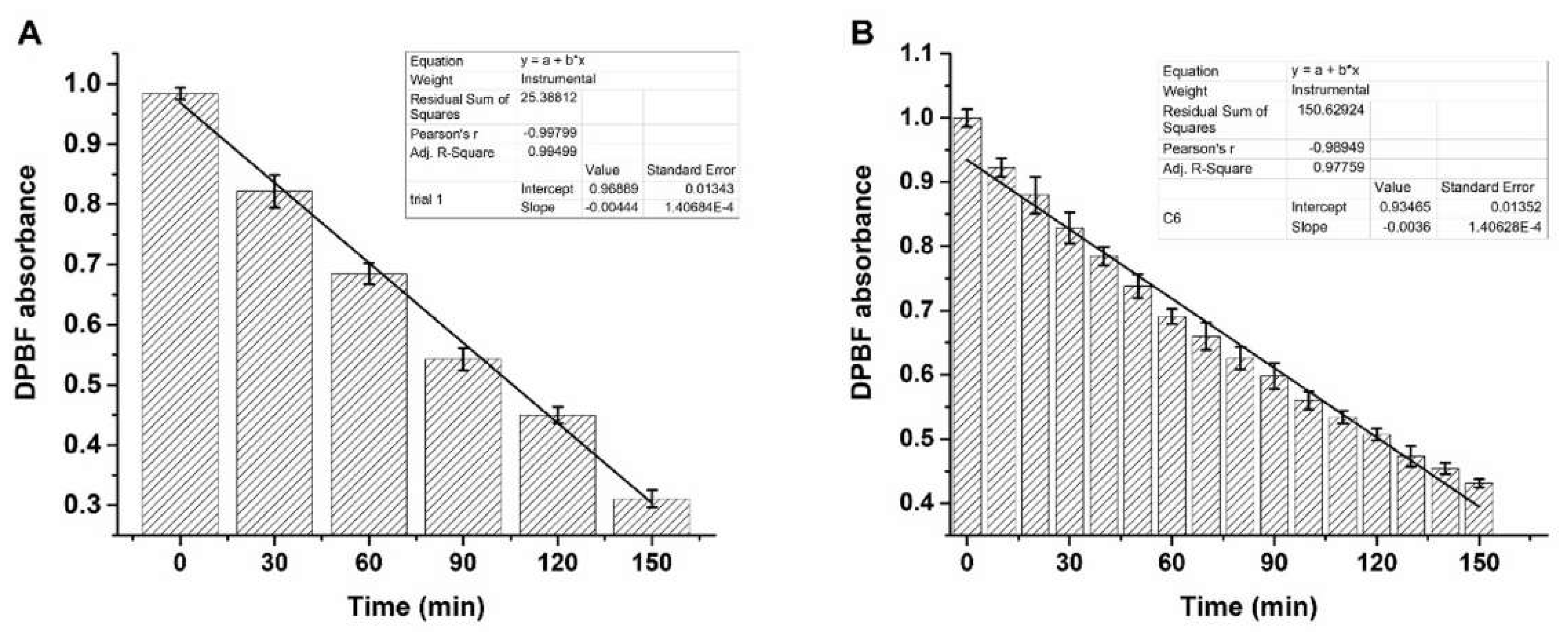

Supplement: Figure S10 — Average decline in DPBF absorbance at 411 nm for Bod PS (A) and Bod-TiO2 (B) presented as mean values ± standard deviation; n = 3. [file tjc-47-06-1407s10.tif]

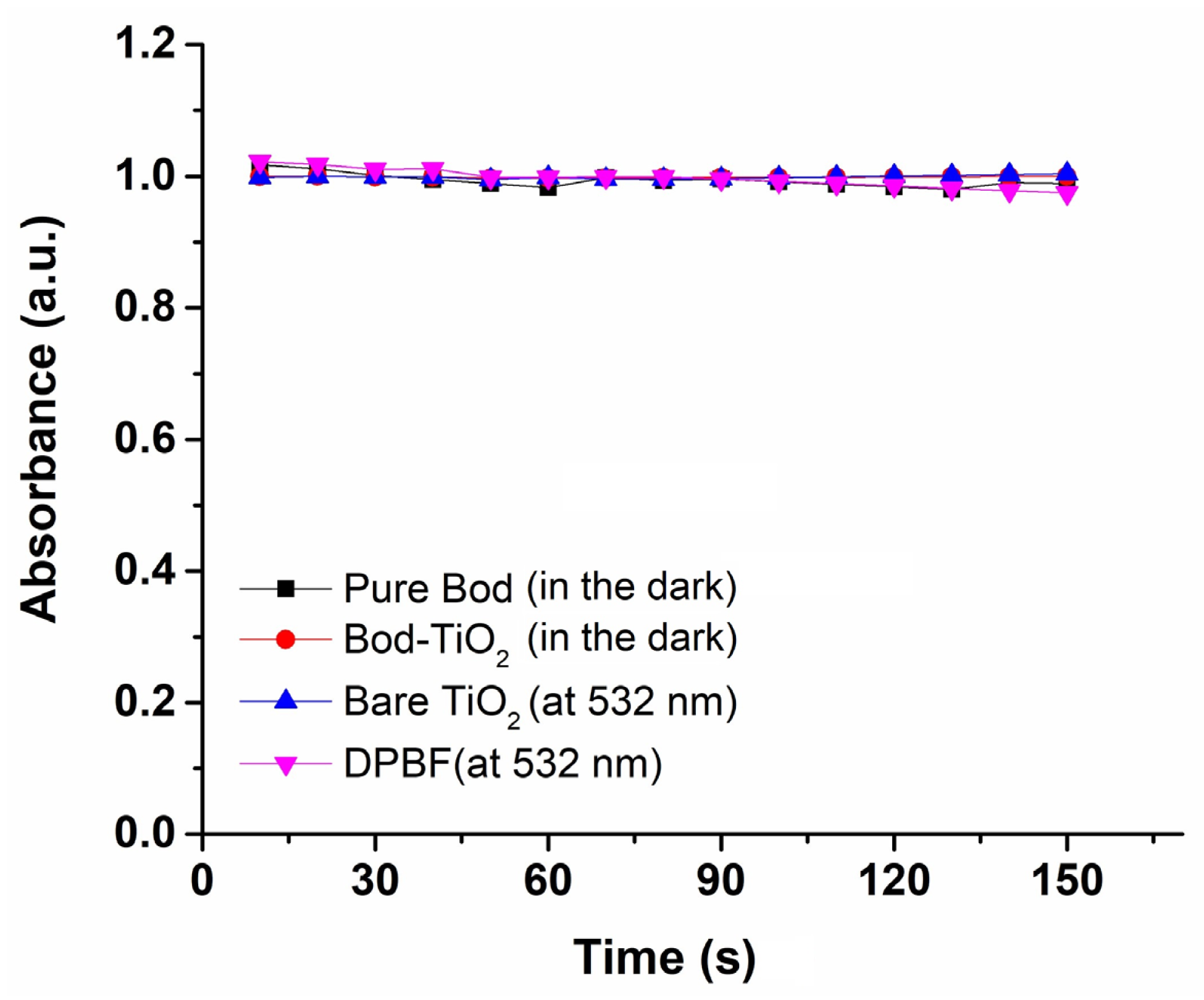

Supplement: Figure S11 — Control experiments: UV-Vis spectra of pure Bod, Bod-TiO2, bare TiO2, and DPBF. [file tjc-47-06-1407s11.tif]
